# SANI-Severe Asthma Network in Italy: a way forward to monitor severe asthma

**DOI:** 10.1186/s12948-017-0065-4

**Published:** 2017-04-10

**Authors:** G. Senna, M. Guerriero, P. L. Paggiaro, F. Blasi, M. Caminati, E. Heffler, M. Latorre, G. W. Canonica, on Behalf of SANI, Adiletta Girolamo, Bonavia Marco, Bucca Caterina, Caiaffa Maria Filomena, Calabrese Cecilia, Camiciottoli Gianna, Capano Pasquale, Centanni Stefano, Chieco Bianchi Fulvia, Colombo Giselda, Corsico Angelo Guido, Costantino Maria Teresa, Crimi Nunzio, Crivellaro Mariangiola, D’Amato Maria, Del Giacco Stefano, De Paulis Amato, Di Gioacchino Mario, Di Maria Giuseppe, Domenico Schiavino, Favero Elisabetta, Foschino Maria Pia, Girbino Giuseppe, Guarnieri Gabriella, Lombardi Carlo, Macchia Luigi, Maestrelli Piero, Maggi Enrico, Marinari Stefano, Maselli Rosario, Milanese Manlio, Nava Stefano, Papi Alberto, Patella Vincenzo, Pelaia Girolamo, Poto Sergio, Ridolo Erminia, Rolla Giovanni, Rottoli Paola, Santus Pierachille, Savi Eleonora, Schiavino Domenico, Schichilone Nicola, Severino Maurizio Giuseppe, Spadaro Giuseppe, Stanziola Anna, Torchio Roberto, Triggiani Massimo, Vianello Andrea, Zappa Maria Cristina

**Affiliations:** 1 Asthma Center and Allergy Unit, Verona University and General Hospital, Piazzale Stefani 1, 37126 Verona, Italy; 2 Department of Computer Science, University of Verona, Strada Le Grazie, 15, 37134 Verona, Italy; 3 Cardio-Thoracic and Vascular Department, University of Pisa, Via Paradisa, 2, 56124 Pisa, Italy; 4 Department of Pathophysiology and Transplantation, Università degli Studi di Milano, Cardio-thoracic unit and Cystic Fibrosis Adult Center Fondazione IRCCS Cà Granda Ospedale Maggiore Policlinico, Via Francesco Sforza, 35, 20122 Milan, Italy; 5 Respiratory Diseases and Allergy – Department of Clinical and Experimental Medicine, University of Catania, Via Santa Sofia, 78, 95123 Catania, Italy; 6 Allergy & Respiratory Disease, DIMI-University of Genova, Largo Rosanna Benzi, 10, 16132 Genova, Italy; 7 Asthma & Allergy Clinic, Humanitas University, Via manzoni 56, 20089 Rozzano, Milano Italy

**Keywords:** Severe asthma, Network, Registry, Biologics, Adherence

## Abstract

Even if severe asthma (SA) accounts for 5–10% of all cases of the disease, it is currently a crucial unmet need, owing its difficult clinical management and its high social costs. For this reason several networks, focused on SA have been organized in some countries, in order to select these patients, to recognize their clinical features, to evaluate their adherence, to classify their biological/clinical phenotypes, to identify their eligibility to the new biologic therapies and to quantify the costs of the disease. Aim of the present paper is to describe the ongoing Italian Severe Asthma Network (SANI). Up today 49 centres have been selected, widespread on the national territory. Sharing the same diagnostic protocol, data regarding patients with SA will be collected and processed in a web platform. After their recruitment, SA patients will be followed in the long term in order to investigate the natural history of the disease. Besides clinical data, the cost/benefit evaluation of the new biologics will be verified as well as the search of peculiar biomarker(s) of the disease.

## Background

Asthma is a common disease being its prevalence worldwide around 5–7%. Despite effective treatments and management guidelines, 5–10% of asthmatics suffer from severe asthma, characterized by frequent exacerbations, regular use of high dose of inhaled steroids and need of frequent burst of oral steroids, unscheduled visits, accesses to emergency room and hospitalizations [1]. Though the prevalence of severe asthma is relatively low, it accounts for 50% of the global costs of the disease, being a clinical as well as social problem.

Severe asthma is an heterogeneous disease, with different clinical and inflammatory phenotypes [2, 3]. This large heterogeneity in the clinical and biological manifestations of severe asthma is important for prognosis, but mainly for selecting specific targets for pharmacologic and non pharmacologic interventions, and also for selecting patients potentially moving from severe asthma to more systemic diseases [4].

Besides the pharmacological treatment, currently two biologics are available as add-on therapy for those patients: omalizumab, an anti-IgE monoclonal antibody, and mepolizumab, an anti-IL5 monoclonal antibody, approved and included in GINA Guidelines 2016 [5]. However, several other biologics targeting different cytokines involved in the inflammatory cascade are under evaluation (dupilumab—anti IL4recα-IL13, benralizumab—anti IL5, tralokinumab and lebrikizumab—anti IL13) [6–8]. These drugs will offer a powerful improvement in the management of the disease but, on the other hand, due to their high costs, the sustainability of these treatments is strongly based on strict criteria of patient’s eligibility. Therefore the identification and the accurate selection of patients with SA is currently a crucial unmet need in the management of the disease [9].

To face up to this problem several registries collecting the cases of severe asthma have been create recently in some countries. After the American TENOR cohort study [10, 11], subsequently in Europe similar registries were also developed in Belgium [12], in Spain [13, 14] and also in Italy, though limited to the North–East territory or related to specific research studies [15–17]. Possibly the most well organized registry on refractory asthma has been create in the United Kingdom by the British Thoracic Society [18–22]. Many issues have been addressed by these networks, such as the clinical features and phenotypes of severe asthma, their stability over time, the cost of the disease and of their comorbidity, the efficacy and the effectiveness of the biologic therapy (Table 1).

**Table 1:** Registries on severe asthma currently available in USA and Europe

Registry (reference)	Country	No. of centers	Study population	Outcomes
TENOR [8, 9]	USA	283	4.756	Natural history of SA
BSAR [10]	Belgium	9	350	Definition of clinical phenotypes in SA
Spanish Multi-Centers Registry [11]	Spain	30	266	Omalizumab efficacy
Spanish Multi-Centers Registry [12]	Spain	30	295	Omalizumab efficacy
NEONET [13]	Italy	9	112	Omalizumab efficacy and safety
ARRISA [14]	UK	29 Primary care practices	911	Impact of the network on exacerbation
BTS Severe Refractory Asthma Registry [15]	UK	4	382	Phenotype characterization, standardized assessment
BTS Severe Refractory Asthma Registry [16]	UK	4	349	Three years follow up
BTS Severe Refractory Asthma Registry [17]	UK	4	349	Phenotype stability over time
BTS Severe Refractory Asthma Registry [18]	UK	7	516	Economic analysis of SA
BTS Severe Refractory Asthma Registry [19]	UK	7	808	Comorbidity due to the use of systemic steroid

*TENOR* The epidemiology and natural history of asthma:outcomes and treatment regimens, *BSAR* Belgian Severe Asthma Registry, *NEONET* Italian North-East Omalizumab Network, *ARRISA* at risk register in severe asthma, *BTS* British Thoracic Society

The present paper focuses on the ongoing project of an Italian National Registry, SANI (Severe Asthma Network in Italy) promoted by GINA Italy—SIAAIC (Italian Society Allergy, Asthma and Clinical Immunology) and SIP/IRS (Italian Respiratory Society). The aim of this network is to enroll patients with severe asthma, in a real life setting, recruited by specialized centers, homogeneously placed on a database management system to follow them over the time.

The relevance of the real life studies is particularly critical in SA, where most of clinical data come from large controlled studies. However, as recently emphasized, there is a long distance between the patients strictly selected for clinical trials and the patients usually visited in daily routine. Randomized control trials are the cornerstone of the evidence based medicine as they are designed to evaluate the efficacy and safety of a particular treatment in a population under ideal condition. The aim of the real life studies to assess the real life efficacy of the same treatment, known as “effectiveness” [23, 24]. Recently this gap has been clearly shown as regards omalizumab treatments [25].

## Design and participants

The Italian asthma observatory is a web-based registry encompassing demographic, clinical, functional and inflammatory data of severe asthmatics (SA), recruited by Italian Unit of Allergy and Pulmonology. The web platform has been already successfully tested in a recent previous pilot study [15], and further improved by collecting the suggestions of SANI Scientific Committee members. Under a technical perspective, the platform is conceived in order to facilitate data entry as much as possible. Drop down menus are provided for most of the fields; the follow-up pages are prefilled with the information included at baseline, available for updates only; an automatic follow-up alert system is provided by email to every clinician for each of the patients included. Participant centers have access with a code to include anonymously the data of patients. This step is needed to assure privacy requirements despite data sharing. However the matching with other national registries of interests (i.e. hospitalizations for asthma exacerbations, pharmaceutical data and so on) will be possible as the coding system is completely tracked and accessible by the platform technical staff.

The information collected will provide:

- The collection of homogeneous clinical, functional and biologic data of patients with SA in a real life setting.
- The evaluation of adherence to treatment in real life.
- The clinical eligibility of patients treated with biologics.
- The evaluation of patients’ clinical response to each treatment.
- The monitoring of tolerability and safety.
- The long-term follow up of patients with SA.

**Fig. 1:**
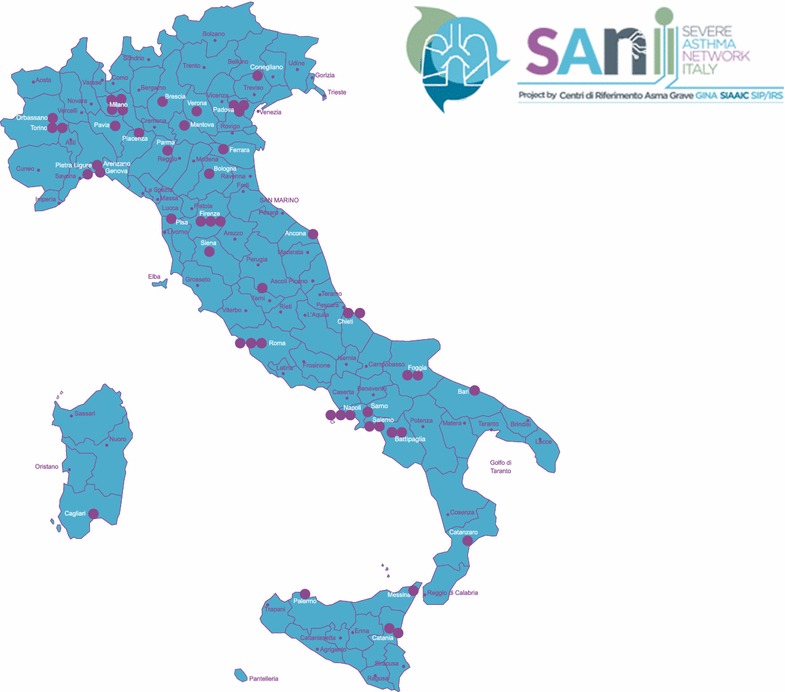
Geographic distribution of Referral Centers currently involved in SANI project

Every center to be included needs to submit an application form in which clinical and scientific issues were evaluated such as personnel dedicated to asthma (specialists and nurses), population of asthmatics yearly treated, availability of lung function equipment and other clinical procedures, number and quality of scientific publications on asthma and severe asthma. For each aspect documentation is required and each item is evaluated by a scoring system validated within the Scientific Committee. The maximum score is 100 points. To be eligible every center has to achieve a minimum score of 75. Up to now all the applicants have reached the minimum threshold and overall 51 centers have been recruited, distributed throughout the Italian territory (Fig. 1).

The patient enrollment protocol has been approved by the Central (P.I.) Ethics Committee, and the enrollment in the other Centers starts upon approval of each Ethics Committee of reference.

## Study population

Inclusion criteria are:

- Age >12 years.
- Diagnosis of SA according to the ERS/ATS criteria [1].
- Lack of asthma control despite regular treatment with the combination of high dosage of inhaled corticosteroids (ICS) and beta 2 long acting.

Exclusion criteria have not been considered in order to have a realistic view of SA in real life. However, as observational studies have suggested that up to 50% of patients referred with severe asthma after a detailed evaluation do not have a refractory disease but different causes responsible for persistent symptoms, a detailed diagnostic protocol will be shared by all participant centers. Furthermore following the first visit patients will be enrolled after a period of 3–5 months of follow up, in order to exclude confounding factors such as a poor adherence to the treatment or the existence of exacerbating factors (passive or active smoking, occupational irritants or allergens etc.).

In the web registry all data collected at the enrollment visit as well as those recorded during the follow up visits, scheduled every 6 months, will be included. Data inclusion of patients recruited will be task of the specialist who is responsible for the treatment. However, data manager supervision will be scheduled for each participant center every 3 months.

For each participant the following information will be collected:

1. Demographic data (age, sex, height, weight, BMI).
2. Clinical features (presence of allergies and/or comorbidity, lung function, previous accesses to ER and/or hospitalizations).
3. Asthma control in the previous month according to the GINA Guidelines [5] and standardized questionnaires (ACT, ACQ).
4. Adherence to treatment.
5. Presence of potential future risks.
6. Concomitant regular and on demand treatments, Including AIT-Allergen Immunotherapy.
7. Treatments used for comorbidities (e.g. steroids for nasal polyposis).
8. Reports of previous adverse reactions to the drugs/biologics used.
9. Inflammatory markers (FeNO, eosinophils in the blood and/or in the sputum).
10. Reasons for withdrawal from biologic treatment.
11. Assessment of the quality of life through standardized questionnaires (AQLQ) which will be drawn up by the patient without the support of any parents, nurses or physicians.

The length of the follow up for each patient is scheduled for 10 years.

All the information listed above will be collected both at baseline and at every follow-up visit. Timing and type of requested information represent a specific standardized protocol to be followed by the participating centers in assessing and monitoring recruited patients. It will allow creating a quite homogeneous data set despite the real life setting.

## Statistical analysis

The web application contains a section, which includes the most important statistics and graphs updated in real time for the whole network and for each single participant center. Results are expressed as a mean and standard deviation for continuous variables, as percentage for categorical ones. Shapiro–Wilk test will be used to test the normal distribution of continuous variables. Two-sample test of proportion will be employed to compare two groups for categorical data whereas two sample t test to compare the mean of two groups. Chi square test or Fisher test will be applied to assess the association between categorical variables. Many other statistical multivariate models will be implemented to investigate the relationships between outcome variables and predictors variables. Every statistics will be run in real time on the web-application via R software or Stata software.

The first purpose of the data collection is providing epidemiological end descriptive information about severe asthma in Italy, which is the currently the main unmet need. The above-mentioned statistical analysis lines are already included in the protocol approved by the Central (P.I.) Ethics Committee and they allow expanding the research lines on our dataset. The Scientific Committee will evaluate any application for further analysis proposals, and if needed amendments to the main protocol will be submitted to the Ethics Committees.

## Ethical issues

The observatory will be carried out according to the declarations of Helsinki and Oviedo. The protocol has been performed according to the principles and procedures of the Good Clinical Practice (ICH Harmonized Tripartite Guidelines for Good Clinical Practice 1996; Directive 91/507. EEC, The Rules Governing Medical Products in the European Community) and in accordance with the Italian laws (D.L.vo n.211 del 24 Giugno 2003; D.L. n.200 del 6 Novembre 2007; MD del 21 Dicembre 2007). SANI initiative is supported by several pharmaceutical companies, which have been listed in the acknowledgement. They provide unrestricted grants and they do not have any role in the study design, planned analysis. In fact they are not having representatives in the scientific committee.

## Concluding remarks

“Precision medicine” is now the new challenge in asthma treatment. This approach is related to the growing evidence of different asthma phenotypes and endotypes, which may require specific approaches to the pharmacologic treatments. This is particularly true for severe uncontrolled asthma, where new expensive biologic drugs will be available in the next future. Therefore, an accurate assessment of these patients and the collection of a large database in our country are compulsory for having a greater knowledge of this subgroup of asthmatic patients.

The SANI project would like to cover this gap of accurate characterization of patient affected by severe uncontrolled asthma, in order to promote an appropriate assessment and therapeutic management of these complex patients.

## Key points

- Severe asthma still represents an unmeet need in the asthma management and its epidemiologic burden as well as its clinical classification are still unclear.
- In order to address these problems it is critical to select patients according to strict diagnostic criteria and consequently to collect a large homogeneous population sample to evaluate.
- Besides the clinical and economic assessment the availability of a large population of SA patients represents the starting point for biomarker research, aimed to identify in advance the potential responder and not responder to single biologic treatment. This finding will be the cornerstone for the future financial sustainability of these high cost treatments [8, 9, 26].
- Long term follow up will include monitoring of treatment safety and tolerability.
- Long term follow up will also provide possible data concerning the persistence of effectiveness of treatment after suspension.

## Abbreviations

ATS: American Thoracic Society
ACT: Asthma Control Test
ACQ: Asthma Control Questionnaire
AQLQ: Asthma Quality Of Life Questionnaire
AIT: allergen immunotherapy
BMI: body mass index
ERS: European Respiratory Society
GINA: global initiative for asthma
ICS: inhaled corticosteroids
PI: principal investigator
SA: severe asthma
SIAAIC: Italian Society Allergy, Asthma and Clinical Immunology
SIP/IRS: Italian Respiratory Society
